# Genomic stratification based on microenvironment immune types and PD-L1 for tailoring therapeutic strategies in bladder cancer

**DOI:** 10.1186/s12885-021-08350-1

**Published:** 2021-05-31

**Authors:** Xintong Lyu, Ping Wang, Qiao Qiao, Yuanjun Jiang

**Affiliations:** 1 Department of Radiotherapy, First Hospital of China Medical University, Shenyang, Liaoning China; 2 Department of Urology, First hospital of China Medical University, Shenyang, Liaoning China

**Keywords:** Immuno-oncology, Genomics, Immune subtypes, Bladder cancer, Therapeutic strategies

## Abstract

**Background:**

The tumour microenvironment (TME) not only plays a role during tumour progression and metastasis but also profoundly influences treatment efficacy. Environment-mediated drug resistance is a result of crosstalk between tumour cells and stroma. The presence of a “stromal exhaustion” response is suggested by the T cell exhaustion signature and PD-L1 expression. The prognostic role of PD-L1 in bladder cancer has been investigated in previous studies, but the results remain inconclusive. For a more comprehensive study, we discuss potential strategies to improve effectiveness in patients with various TME statuses and PD-L1 expression levels.

**Methods:**

We estimated the prognostic role of PD-L1 using immunohistochemistry and identified four immune subtypes according to the type of stromal immune modulation and PD-L1 expression status.

**Results:**

Patients in the PD-L1-low-exhausted group had the worst prognosis and showed the worst antigen-presenting cell (APC) immunosuppression status. The PD-L1-low-exhausted group showed the highest amount of infiltration by macrophage M2 cells, naïve B cells and resting mast cells. The TMB and the effectiveness of anti-PD-1 treatment were significantly increased in the PD-L1-high expression groups compared with the PD-L1-low expression groups. In the PD-L1-high groups, patients who underwent chemotherapy exhibited better overall survival rates than patients who did not undergo chemotherapy, whereas there was no significant difference in the PD-L1-low groups. We performed gene set enrichment analysis (GSEA) to explore the critical pathways that were active in the PD-L1-low-exhausted group, including the myogenesis, epithelial-mesenchymal transition and adipogenesis pathways. Copy number variations (CNVs) were related to the expression levels of differentially expressed genes upregulated in the PD-L1-low-exhausted group, including LCNL1, FBP1 and RASL11B. In addition, RASL11B played a role in predicting overall survival according to The Cancer Genome Atlas data, and this finding was validated in the PD-L1-low-exhausted group in the Gene Expression Omnibus database (GEO).

**Conclusion:**

The immune environment of tumours plays an important role in the therapeutic response rate, and defining the immune groups plays a critical role in predicting disease outcome and strategy effectiveness.

## Background

Bladder cancer is the fifth most common cancer and is diagnosed in greater than 430,000 patients every year worldwide [1]. Bladder cancer shows a sex bias with approximately 75% of patients being male and ranks fourth in incidence and eighth in mortality among cancers in males [2, 3]. Approximately 20% of bladder cancer patients are diagnosed with muscle-invasive bladder cancer (MIBC). Treatment options include radical cystectomy and chemotherapy, but the prognosis of patients with metastasis or nonorgan confinement can be poor [4].

Recently, immune checkpoint blockade has changed the therapy landscape in bladder cancer. Currently, anti-PD-1/PD-L1 strategies have been recommended as the first-line treatment for cisplatin-intolerant PD-1-positive patients with local late/metastatic bladder cancer (2020 NCCN v2). After several decades with no significant advanced therapeutic measures, some clinical trials have demonstrated that durable effects can be achieved in 15–25% cisplatin-resistant metastatic urothelial cancer (UC) patients treated with PD-1/PD-L1 antibodies [5–10]. Given that only a few patients benefit from PD-1/PD-L1 antibodies, there remains a critical need to explore mechanisms of resistance [11].

The tumour microenvironment (TME) comprises immune cells, endothelial cells and cytokines, growth factors, etc. The TME not only plays a role during tumour progression and metastasis but also has profound influences on the efficacy of treatment. Environment-mediated drug resistance is a result of crosstalk between tumour cells and stroma [12]. “Hot tumours” filled with immune cells are often considered to be more sensitive to immunotherapy than “cold tumours”, which have fewer immune cells. The role of tumour-infiltrating lymphocytes (TILs) and cytokines in immunotherapy has been demonstrated [13]. Nevertheless, the effect of the TME on the response to immunotherapy remains controversial in urothelial carcinoma [14]. Efforts are currently focused on investigating the relationships between molecular subtype and immunotherapy response. Recently, the TCGA molecular subtype (k = 5) classification was applied to the IMvigor 210 atezolizumab trial data. The results indicated that the neuroendocrine-like (NE-like) and luminal subtypes were both associated with high response rates [15]. However, the opposite pattern of NE-like tumour response to immunotherapy was observed with the Decipher classifier [16]. Therefore, further characterization of the immune profile to identify subtypes in bladder cancer that may be relevant for therapy choice is essential.

In this study, to identify common immune subgroups, evaluate how the immune components of the TME regulate therapeutic effectiveness and explore potential strategies to improve the effectiveness of patients with various immune statuses in bladder cancer, we divided patients into four subgroups based on a combination of PD-L1 expression and the type of immune modulation, and then we performed a series of analyses. First, survival analysis was performed to assess the associations between immune subgroups and patient prognosis. We then compiled published tumour immune expression signatures and obtained scores for all samples. The leukocyte fraction, predicted neoantigens, proliferation rate, B cell receptor (BCR) expression, cancer testis antigen (CTA) expression, immunomodulator (IM) expression and DNA damage were characterized in the samples. In addition, we next estimated the mean fractions of immune cells in samples to assess the relationship between immunogenicity and immune infiltration. Finally, we sought to identify the underlying molecular mechanism governing the various immune responses across subgroups. GSEA at the RNA level and CNV analysis at the DNA level were performed to identify the key pathways and key genes in the PD-L1-low-exhausted group and provide potential strategies for patients with various immune statuses.

## Methods

### Data processing

Within the TCGA database, we assessed 363 bladder cancer samples with mRNA expression data, CNV data and clinical data. We limited our research to data for samples with the “primary tumour” sample type (*n* = 357) and excluded samples with the “solid normal tissue” sample type (*n* = 6). For the tumour mutation burden (TMB), we obtained data from a previously published signature [17]. The normalized level three RNA sequencing data, CNV data and clinical data were downloaded from https://genome-cancer.ucsc.edu/.

### Immunohistochemistry

A total of 106 biopsy samples from patients with primary bladder cancer were collected at the First Hospital of China Medical University between 2009 and 2012. All the patients accepted radical surgery. The primary antibody was PD-L1 (mouse anti-human PD-L1 monoclonal, dilution 1:100, Abcam). The percentage of positive cells and staining intensity were estimated. Consistent with previous reports, staining in greater than 5% of the tumour cells was considered positive.

### Clustering & definition of groups

In previous studies, “activated” stroma was characterized by an activated fibroblast state, a set of genes associated with macrophages, and the expression of other genes that point to its role in tumour promotion. The “normal” stromal factor may describe a “good” version of the stroma, whereas the “activated” stromal factor may describe an activated inflammatory stromal response to be responsible for disease progression [18]. Based on the expression of the “activated” stroma gene signature [19], we divided patients into “activated” (“normal” stroma) and “exhausted” (“activated” stroma) subgroups using the consensus clustering algorithm (k = 2). Consensus clustering of the samples using “activated” stroma genes was refined using a random forest (RF) algorithm developed under the ConsensusClusterPlus R package. To investigate the clinicopathological significance of PD-L1 expression and the type of immune modulation, the cohort was divided into four subgroups: (1) “PD-L1-high-activated”, (2) “PD-L1-high-exhausted”, (3) “PD-L1-low-activated” and (4) “PD-L1-low-exhausted”.

### Gene set enrichment analysis (GSEA)

GSEA was performed using a Java GSEA desktop application (www.broad.mit.edu/gsea/). The hallmark gene set “h.all.v7.0.symbols.gmt” was used in this study. A nominal *P*-value < 0.05 and a false discovery rate (FDR) < 0.25 were used to determine significance.

### GEO validation dataset

Validation data (GES48277) were downloaded from the GEO database (www.ncbi.nlm.nih.gov/geo), and we limited our research to bladder tumour biopsy sample data. We divided patients into four subgroups according to PD-L1 expression and the type of immune modulation. To validate the role of RASL11B, we evaluated its expression in the subgroups.

### Statistical analysis

We identified differentially expressed genes (DEGs) based on the criteria of a false discovery rate < 0.05 and a log fold-change > 1.0, and we performed copy number variation (CNV) analysis with the criterion of a *P*-value < 0.05 and estimated the infiltration of immune cells using R 3.6.1. We adopted an immunophenoscore (IPS) from The Cancer Immunome Atlas, which was based on the expression of the representative genes or gene sets comprising four categories: MHC molecules, immunomodulators, effector cells (CD8+ T cells and CD4+ T cells), and suppressor cells (Tregs and MDSCs). The IPS was indicated to be a superior predictor of response to anti-PD-1 antibodies in two independent cohorts [20]. We adopted chemosensitivity [21] and immune expression signatures from a previously published signature [22]. The χ2 test was employed to compare the clinical and pathological characteristics of the groups. Data distributions were tested using the Kolmogorov-Smirnov test. Parametric or nonparametric tests were employed according to the results. Immune expression signatures, DNA damage signatures and gene expression of IMs were compared among subgroups using ANOVA for continuous variables. Kaplan–Meier analysis for the overall survival of patients in TCGA dataset was performed using the GEPIA web server [23]. Two-tailed *P*-values less than 0.05 were considered statistically significant. Statistical analyses were performed using IBM SPSS Statistics software version 24 and GraphPad Prism software 7.00.

## Results

### Classification of patients into subgroups according to PD-L1 expression and the type of immune modulation

**Fig. 1:**
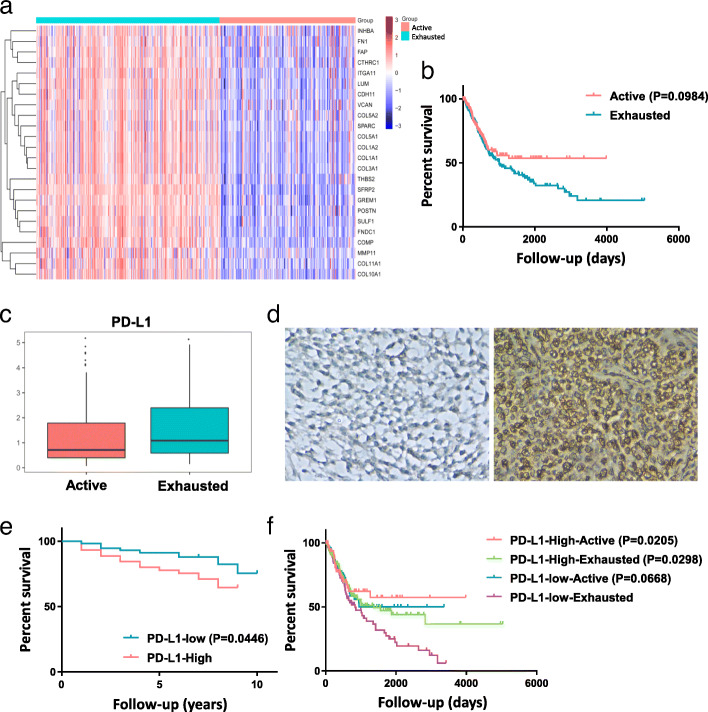
Immune subgroups according to PD-L1 expression and the type of immune modulation. **a** Immune modulation in the response of immune subgroups is indicated by the heatmap. Each row indicates a gene, and each column indicates a sample. **b** Kaplan-Meier plot showing that the active (red) and exhausted (blue) groups were not significantly different, *P* = 0.0984. **c** The exhausted group showed higher PD-L1 mRNA expression than the active group (1.54 vs 1.22, *P* = 0.012). **d** Representative immunohistochemical staining of PD-L1. Right: strong PD-L1 expression; left: negative PD-L1 expression. Original magnification: × 400. **e** Kaplan-Meier plot showing that PD-L1 overexpression predicts worse survival outcomes in bladder cancer. **f** Kaplan-Meier plot showing that different immune subgroups had significantly different prognoses

Given that immune responses can both prevent and promote cancer progression [24], the subtype of immune modulation should be considered. Accordingly, an “exhausted” response was suggested to predict worse survival in pancreatic ductal adenocarcinoma [19], whereas there was no significant difference between an “exhausted” (162/363) or an “active” response (201/363) in our study (*P* = 0.09) (Fig. 1a, b). As the presence of an “exhaustion” response was suggested by the T cell exhaustion signature [25], we analysed the PD-L1 mRNA expression level in two subgroups. The exhausted group exhibited increased PD-L1 mRNA expression than the active group (1.54 ± 1.20 vs 1.22 ± 1.16, *P* = 0.012) (Fig. 1c). The prognostic role of PD-L1 in bladder cancer has been investigated in previous studies, but the results remain inconclusive. For a more comprehensive study, we estimated the prognostic role of PD-L1 using immunohistochemistry. Patient biopsy samples (*n* = 106) were assessed for PD-L1 expression in tumour tissues (Fig. 1d). The results suggested that PD-L1 overexpression could predict worse survival outcomes in bladder cancer (*P* = 0.045) (Fig. 1e). Next, we divided patients into four subgroups based on a combination of PD-L1 expression and the type of immune modulation, and the Kaplan-Meier curves for overall survival are shown in Fig. 1f. The PD-L1-low-exhausted group had a worse prognosis compared with the other groups. We next investigated the clinical characteristics across subgroups and PD-L1 expression levels. The subgroups showed similar clinical characteristics. In addition, previously published bladder cancer molecular subtypes were analysed (Table 1). The basal and luminal subtypes were divided by TCGA into luminal-papillary, luminal-infiltrated, luminal, basal-squamous and neuronal subtypes [26]. We detected the highest proportion of the luminal papillary subgroup within the PD-L1-low-activated group (chi-square, 80.9%, *P* < 0.0001), whereas the basal squamous subgroup harboured the highest proportion of the PD-L1-high-activated and PD-L1-high-exhausted groups (chi-square, 49.3 and 67.6%, *P* < 0.0001). It has been suggested that the luminal-infiltrated subtype exhibited increased PD-L1 and PD-1 expression [26]. Interestingly, we found that approximately 53.1% of luminal-infiltrated subtype samples belong to the PD-L1-low-exhausted group and 37.5% of luminal-infiltrated subtype samples belong to the PD-L1-high-exhausted group, suggesting that a portion of the luminal-infiltrated subtype with decreased PD-L1 expression may exhibit poor anti-PD-1 treatment effectiveness.

**Table 1:** Clinicopathological features of subgroups

Variable	Subgroup				P-value
	PD-L1-high-actived	PD-L1-high-Exhausted	PD-L1-low-actived	PD-L1-low-Exhausted	
Age	64.43 (11.24)	68.37 (10.05)	64.89 (9.75)	71.84 (9.36)	< 0.0001
BMI	26.98 (5.29)	27.31 (5.79)	26.44 (5.80)	26.90 (5.73)	< 0.0001
Sex					0.567
female	16 (23.9%)	33 (29.7%)	20 (21.3%)	22 (25.9%)	
male	51 (76.1%)	78 (70.3%)	74 (78.7%)	63 (74.1%)	
Race					< 0.0001
Asian	6 (9.0%)	6 (5.4%)	24 (25.5%)	6 (7.1%)	
Black	2 (3.0%)	7 (6.3%)	5 (5.3%)	3 (3.5%)	
White	56 (83.6%)	96 (86.5%)	57 (60.6%)	73 (85.9%)	
Not report	3 (4.5%)	2 (1.8%)	8 (8.5%)	3 (3.5%)	
Lymphovascular					0.106
Yes	26 (38.8%)	39 (35.1%)	26 (27.7%)	41 (48.2%)	
No	22 (32.8%)	41 (36.9%)	32 (34.0%)	20 (23.5%)	
Not report	19 (28.4%)	31 (27.9%)	36 (38.3%)	24 (28.2%)	
Stage					< 0.0001
Stage I	1 (1.5%)	0 (0%)	2 (2.1%)	0 (0%)	
Stage II	28 (41.8%)	24 (21.6%)	47 (50.0%)	15 (17.6%)	
Stage III	19 (28.4%)	46 (41.4%)	22 (23.4%)	33 (38.8%)	
Stage IV	18 (26.9%)	41 (36.9%)	22 (23.4%)	37 (43.5%)	
Not report	1 (1.5%)	0 (0%)	1 (1.1%)	0 (0%)	
CMC subtype					< 0.0001
Basal squamous	33 (49.3%)	75 (67.6%)	6 (6.4%)	8 (9.4%)	
Luminal	2 (3.0%)	3 (2.7%)	3 (3.2%)	17 (20.0%)	
Luminal infiltrated	4 (6.0%)	24 (21.6%)	2 (2.1%)	34 (40.0%)	
Luminal papillary	23 (34.3%)	7 (6.3%)	76 (80.9%)	23 (27.1%)	
Neuronal	5 (7.5%)	2 (1.8%)	7 (7.4%)	3 (3.5%)	
PD-L1	2.29 (1.11)	2.32 (1.09)	0.47 (0.25)	0.56 (0.25)	< 0.0001

a. ANOVA; b. Chi-square test (and Fisher’s exact test when appropriate)

Abbreviations: *Lymphovascular* lymphovascular invasion present

### The immune states, fraction of infiltrating immune cells and chemosensitivity of the four subgroups

**Fig. 2:**
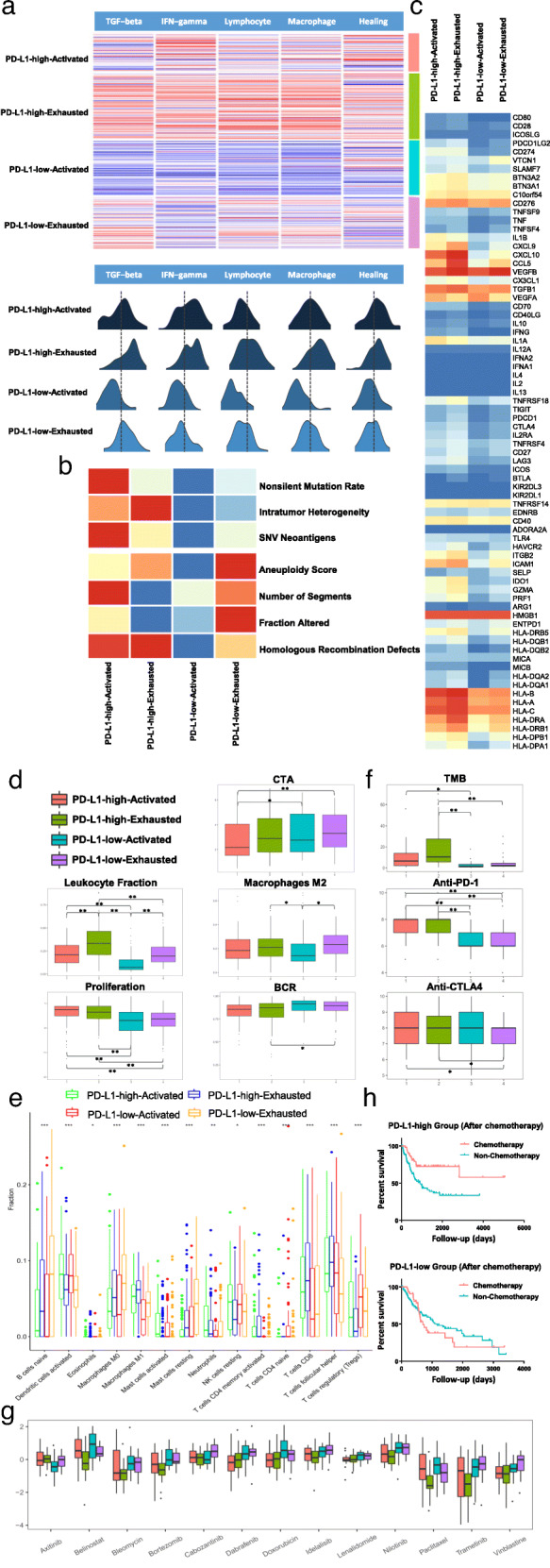
The immune states, fraction of infiltrating immune cells and chemosensitivity in the immune subgroups. **a** The top five modules of the immune subgroups are indicated by the heatmap. The distributions of signature scores within the immune subtypes (rows) are shown, and the dashed line indicates the median. **b** Correlation of DNA damage (rows) with immune subtype. **c** mRNA expression (median normalized expression levels) for 75 IM genes by immune subtype. **d** Values of key immune characteristics by immune subtype. **e** Infiltration of immune cells by immune subgroup. **f** TMB and immunophenoscore (IPS) by immune subgroup. **g** Chemosensitivity by immune subgroup. **h** Kaplan-Meier plot showing that patients who underwent chemotherapy and patients who did not undergo chemotherapy had significantly different prognoses in the PD-L1-high and PD-L1-low subgroups. **i** Crosstalk factor expression (median normalized expression levels) by immune subtype. *P < 0.05; **P < 0.01

Five immune expression signatures were selected to analyse the characteristics of the immune subgroups. The four immune subgroups were characterized by different immune signatures and DNA damage (Fig. 2a, b) and showed distinct immune characteristics (Fig. 2d).

The measures of DNA damage included copy number variation (CNV) burden (including number of segments and fraction of genome alterations), aneuploidy, homologous recombination deficiency (HRD), and intratumour heterogeneity (ITH). Antigen-specific BCR repertoires are critical for the recognition of tumour cells and reflect a robust antitumour response. Gene expression of IMs is critical for immunotherapy of tumours with many ongoing IM agonists and antagonist clinical trials. The leukocyte fraction (LF) was defined as the total leukocyte infiltration [22].

The PD-L1-high-actived group had the highest proliferation rate (Fig. 2d), the lowest CTA scores and high IFN-γ signature (vs. PD-L1-high-exhausted, *P* = 0.235; vs. PD-L1-low-actived, *P* < 0.001; vs. PD-L1-low-exhausted, *P* < 0.001), TGF-β signature (vs. PD-L1-high-exhausted, *P* < 0.001; vs. PD-L1-low-actived, *P* < 0.001; vs. PD-L1-low-exhausted, *P* < 0.001), lymphocyte infiltration (vs. PD-L1-high-exhausted, *P* < 0.001; vs. PD-L1-low-actived, *P* < 0.001; vs. PD-L1-low-exhausted, *P* = 0.583), and macrophage regulation (vs. PD-L1-high-exhausted, *P* < 0.001; vs. PD-L1-low-actived, *P* < 0.001; vs. PD-L1-low-exhausted, *P* = 0.538) and wound healing signatures (vs. PD-L1-high-exhausted, *P* = 0.750; vs. PD-L1-low-actived, *P* < 0.001; vs. PD-L1-low-exhausted, *P* < 0.001). This group displayed the highest number of SNV neoantigens and the highest nonsilent mutation rate.

The PD-L1-high-exhausted group exhibited the highest IFN-γ signature (vs. PD-L1-low-actived, *P* < 0.001; vs. PD-L1-low-exhausted, *P* < 0.001), TGF-β signature (vs. PD-L1-low-actived, *P* < 0.001; vs. PD-L1-low-exhausted, *P* < 0.001), lymphocyte infiltration (vs. PD-L1-low-actived, *P* < 0.001; vs. PD-L1-low-exhausted, *P* < 0.001), macrophage regulation (vs. PD-L1-low-actived, *P* < 0.001; vs. PD-L1-low-exhausted, *P* < 0.001) and wound healing signatures (vs. PD-L1-low-actived, P < 0.001; vs. PD-L1-low-exhausted, *P* < 0.001). This group also exhibited the highest leukocyte fraction and a high number of SNV neoantigens.

The PD-L1-low-actived group was defined by the lowest leukocyte fraction and IFN-γ signature (vs. PD-L1-low-exhausted, *P* = 0.040), TGF-β signature (vs. PD-L1-low-exhausted, *P* < 0.001), lymphocyte infiltration (vs. PD-L1-low-exhausted, *P* < 0.001), macrophage regulation (vs. PD-L1-low-exhausted, *P* < 0.001) and wound healing signatures (vs. PD-L1-low-exhausted, *P* < 0.230). In addition, this group exhibited the highest B cell receptor (BCR) expression, lowest aneuploidy score and lowest number of homologous recombination defects.

The PD-L1-low-exhausted group displayed the highest macrophage M2 infiltration, CTA scores, a low-to-moderate proliferation rate, IFN-γ signature, TGF-β signature, lymphocyte infiltration, macrophage regulation and wound healing signatures.

Gene expression of IMs (Fig. 2c) varied across immune subgroups. The top-ranked genes with differences between subgroups included CXCL9, CXCL10, CCL5, TNFRSF18, ITGB2, ICAM1 and all the HLA genes (consistent with their known interferon inducibility), which were most highly expressed in the PD-L1-high-exhausted group (*P* < 0.001).

We next estimated the mean fractions of immune cells in the four subgroups (Fig. 2e). The PD-L1-high-actived and PD-L1-high-exhausted groups exhibited the highest infiltration of M1 macrophages and CD8 T cells (*P* < 0.001). The PD-L1-high-actived and PD-L1-low-actived groups showed the highest infiltration of activated DCs (*P* < 0.001), whereas the PD-L1-low-actived group exhibited the highest infiltration of Tregs (*P* < 0.001). The PD-L1-low-exhausted group showed the highest infiltration of naïve B cells and resting mast cells, whereas the lowest number of activated mast cells and follicular helper T cells was also observed in this group (*P* < 0.001).

The TMB was significantly higher in the PD-L1-high expression groups compared with the PD-L1-low expression groups [15.06 (PD-L1-high-actived) and 22.32 (PD-L1-high-exhausted) vs. 2.87 (PD-L1-low-actived) and 5.35 (PD-L1-low-exhausted), *P* < 0.001], which confirmed that the effectiveness of anti-PD-1 treatment in the PD-L1-high expression groups [7.64 (PD-L1-high-actived) and 7.69 (PD-L1-high-exhausted) vs. 6.55 (PD-L1-low-actived) and 6.64 (PD-L1-low-exhausted), *P* < 0.001] could be related to TMB (Fig. 2f). In addition, anti-CTLA4 treatment showed no significant differences among the four subgroups.

Recently, stromal immunotypes were demonstrated to be a predictive model to identify patients who would benefit from chemotherapy in bladder cancer [27]. We next estimated the chemosensitivity in the four subgroups. The log-transformed IC50 values are shown in Fig. 2g. Most drugs showed the highest sensitivities in the PD-L1-high-exhausted group, including nilotinib, belinostat, paclitaxel, vinblastine, doxorubicin, trametinib, bortezomib, idelalisib, cabozantinib, and bleomycin. Dabrafenib and lenalidomide showed the highest sensitivities in the PD-L1-high-activated group. Only axitinib showed sensitivity in the PD-L1-low-activated and PD-L1-low-exhausted groups. In addition, in the PD-L1-high groups, patients who underwent chemotherapy exhibited better overall survival rates compared with patients who did not undergo chemotherapy (*P* = 0.0005), whereas there was no significant difference in the PD-L1-low groups (*P* = 0.6066) (Fig. 2h). Finally, to elucidate the crosstalk between tumour cells and immune cells, we estimated the expression of several factors. FGFR3, which supported cancer-associated fibroblast (CAF) survival and activation, exhibited significantly higher expression in the PD-L1-low-actived and PD-L1-low-exhausted groups compared with the other groups. CXCL12, which led to PI3K and ERK1/2 activation in tumour cells, and CCL2, which was released by stromal cells and tumour cells to recruit myeloid cells, exhibited significantly increased expression in the exhausted groups than in the active groups (Fig. 2i) [12].

### GSEA to predict key pathways

**Fig. 3:**
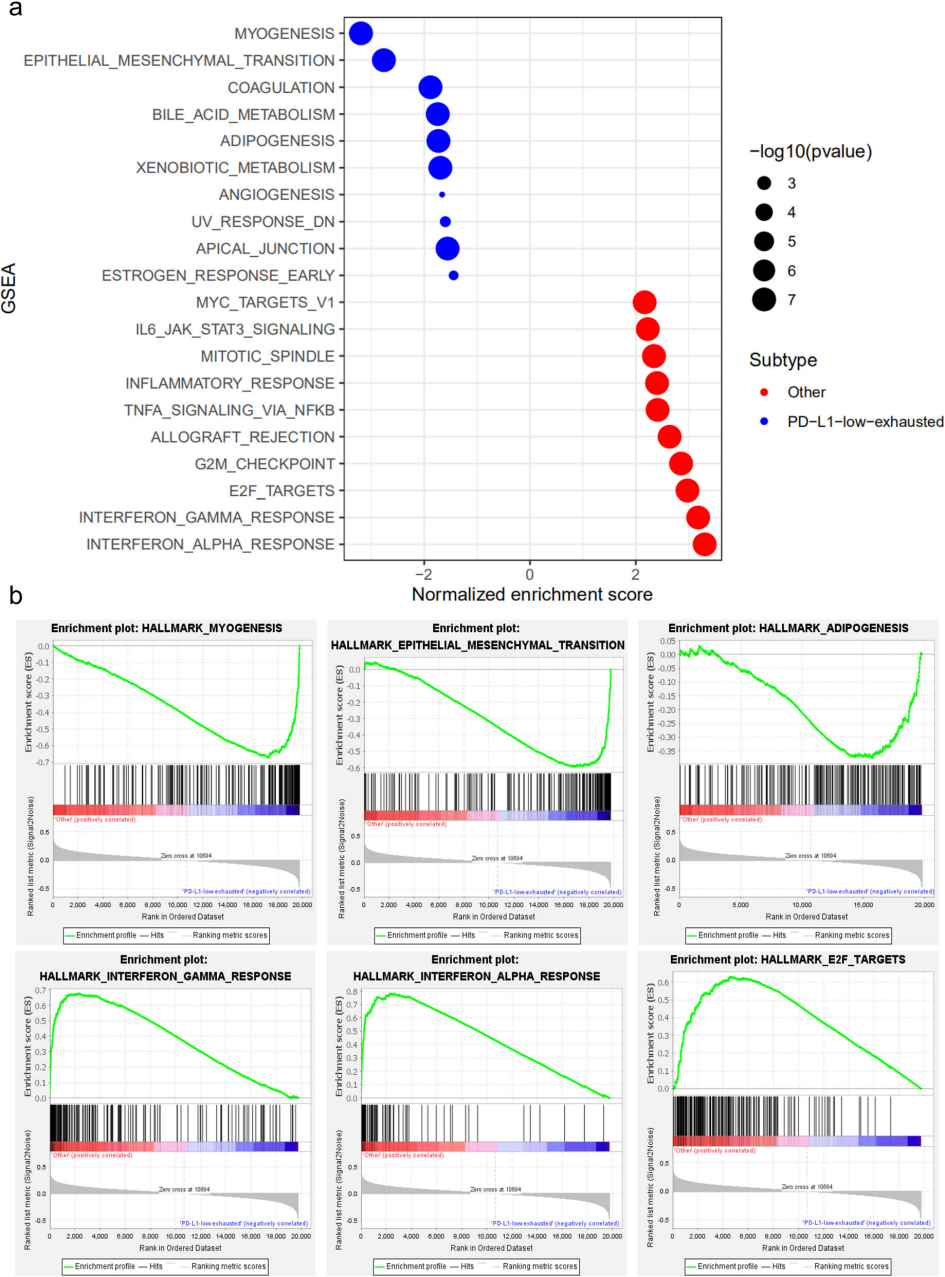
GSEA immune subgroups. **a** GSEA results are indicated by bubbles between the PD-L1-low-exhausted group and the other groups. **b** Key pathways in the two groups

We performed GSEA to explore the critical pathways in the PD-L1-low-exhausted group (Fig. 3a). The upregulated mRNAs were enriched in the myogenesis (normalized enrichment score [NES] = 3.196, nominal *P*-value [NOM *P*-val] < 0.0001, enrichment score [ES] = 0.675), epithelial-mesenchymal transition (NES = 2.763, NOM *P*-val < 0.0001, ES = 0.594) and adipogenesis (NES = 1.731, NOM *P*-val < 0.0001, ES = 0.375) pathways. The downregulated mRNAs were enriched in the interferon alpha response (NES = − 3.302, NOM *P*-val < 0.0001, ES = − 0.784), interferon gamma response (NES = − 3.179, NOM *P*-val < 0.0001, ES = − 0.678) and E2F target (NES = − 2.977, NOM *P*-val < 0.0001, ES = − 0.630) pathways (Fig. 3b).

### Exploring therapeutic targets through CNV analysis

**Fig. 4:**
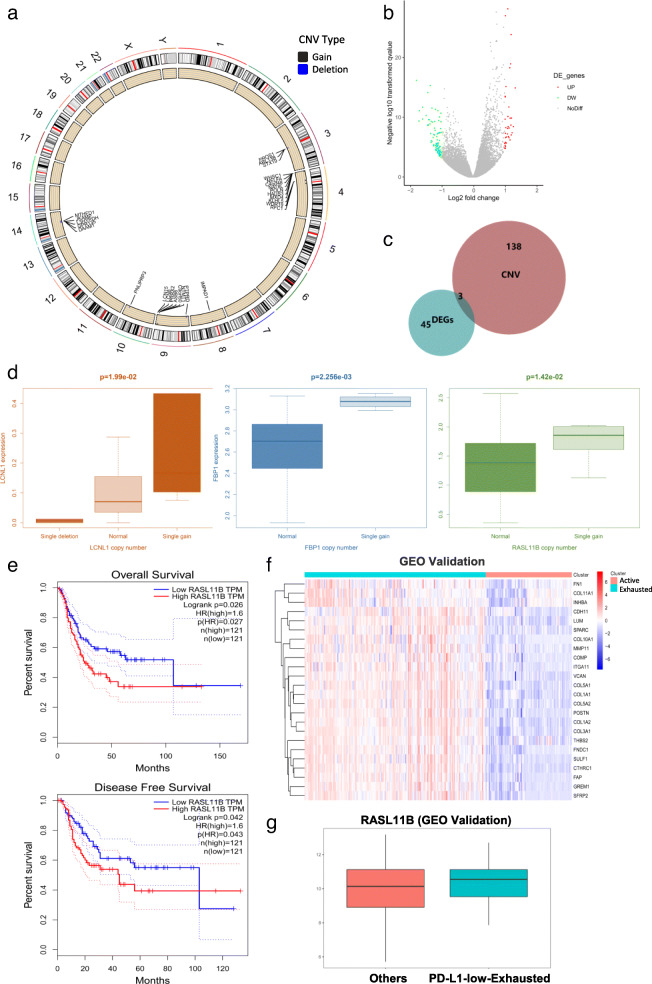
CNVs and DEGs associated with mRNA expression levels and correlated with the overall survival of bladder cancer patients. **a** Differential CNVs between the PD-L1-low-exhausted group and the other groups. Circle: Differential CNV-associated genes in samples according to their chromosomal location. Genes that were gained are labelled in black, and genes that were deleted are labelled in blue. **b** DEGs between the PD-L1-low-exhausted group and the other groups. **c** Venn diagram of the DEGs and CNVs associated with mRNA expression levels. **d** Correlation between different CNV patterns and mRNA expression levels. **e** Kaplan-Meier plots of key genes. **f**. Heatmap depicting immune modulation in the immune subgroups in the GEO validation database. Each row indicates a gene, and each column indicates a sample. **g** RASL11B expression was significantly upregulated in the PD-L1-low-exhausted group compared with the other groups in the GEO validation database

We evaluated the CNV patterns that occurred in the PD-L1-low-exhausted group but not in the other groups. The total number of different CNV events in the PD-L1-low-exhausted group was 381 (*P* < 0.05), and the CNV events mainly included losses of chromosomes 3, 10 and 14 and gains of chromosomes 4 and 9 (*P* < 0.01) (Fig. 4a). Then, we assessed the relationship between CNVs and mRNA expression levels. The results revealed that 141 CNV-driven changes in mRNA expression of the 381 CNVs were associated with the PD-L1-low-exhausted group. To identify the expression characteristics of the PD-L1-low-exhausted group, we further investigated DEGs (Fig. 4b). A total of 6.25% (3/48) of the DEGs associated with the 141 CNVs were related to the expression level of PD-L1, including LCNL1, FBP1 and RASL11B (Fig. 4c, d). In addition, Kaplan-Meier survival curves showed that RASL11B was significant for predicting patient overall survival and disease-free survival (*P* = 0.026 and 0.042, respectively) (Fig. 4e).

Through analysis of the GEO validation data, we found that RASL11B (PD-L1-low-exhausted group: 10.35 ± 1.12; others: 9.99 ± 1.47; *P* = 0.017) showed higher expression in the PD-L1-low-exhausted group (*n* = 119) compared with the other groups (*n* = 182), which was comparable to the results in TCGA dataset.

## Discussion

It is well established that immune status can both support and obstruct tumour progression and therapeutic efficacy [12]. Recently, Richard A. Moffitt et al. published a genetic “active stromo” signature and suggested that patients with an activated stroma had worse survival than patients with a normal stroma. This allowed us to explore stroma-specific subgroups with prognostic and biologic relevance [19]. Tumour-associated macrophages (TAMs) are key regulators of the therapeutic response that produce several suppressive cytokines, thus contributing to T cell suppression. As a key factor of another path to T cell suppression, PD-L1 had different levels between the activated and exhausted groups. To explore the interaction of the two elements, we divided patients into four subgroups based on a combination of PD-L1 expression and the type of immune modulation. Interestingly, the PD-L1-low-exhausted group showed a worse prognosis than the other groups.

To analyse the characteristics of our immune subgroups, five immune expression signatures were selected, and the four subgroups were characterized by distinct immune signatures. The PD-L1-low-exhausted group had the worst prognosis and displayed composite signatures related to a low to moderate proliferation rate, IFN-γ signature, TGF-β signature lymphocyte infiltration, macrophage regulation and wound healing and had the highest M2 macrophage content and CTA scores; these findings are consistent with an immunosuppressed TME, for which a poor outcome would be expected. In contrast, the two subgroups displaying an active immune response (a high IFN-γ signature), the PD-L1-high-actived and PD-L1-high-exhausted group, had the most favourable prognosis, which is consistent with studies suggesting that a type I immune response is needed for antitumour activity [28]. The PD-L1-high-actived group demonstrated the most SNV neoantigens and highest nonsilent mutation rate, in agreement with recent research suggesting that patients with high neoantigen number and high mutation rate exhibit the longest survival in bladder cancer. DNA damaging therapeutics such as radiotherapy and chemotherapy may help liberate neoantigens to induce T cell responses and improve the effects of immunotherapy in the group with the second highest number of SNV neoantigens, the PD-L1-high-exhausted group [26, 29, 30]. The PD-L1-low-actived group was defined by decreased leukocyte chemotaxis, similar to an immunologically quiet subtype [22], leading to fewer immune cells and better outcomes [18].

To further elucidate the mechanism, we estimated the fractions of immune cells in the four subgroups. M1 macrophages and CD8+ T cells highly infiltrated the tumours in the PD-L1-high-actived and PD-L1-high-exhausted groups. It is well established that CD8+ T cells as well as macrophages are required for synergistic curative activity in response to immune checkpoint antibodies (antibodies against CTLA-4, PD-1, and PD-L1) [31]. High PD-L1 levels act as a target to activate high numbers of infiltrating M1 macrophages and CD8+ T cells. In the PD-L1-high subgroups, patients who underwent chemotherapy exhibited better overall survival rates than patients who did not undergo chemotherapy, which may have inhibited high chemosensitivity in these two subgroups.

It should be noted that most of the chemotherapeutic drugs tested in this study had the highest sensitivities in the PD-L1-high-exhausted group, but chemotherapy suppresses the antitumour response of CD8+ T cells [32, 33] and induces IL-10-producing M2 macrophages, which may lead to transformation towards a PD-L1-low-exhausted status [34].

The highest infiltration of Tregs and the lowest infiltration of CD8+ T cells were found in the PD-L1-low-actived group. It was reported that STAT3 inhibition in combination with radiation improved tumour growth delay, decreased Tregs and enhanced effector T cells and M1 macrophages, which may improve curative effects [35]. In addition, the PD-L1-low-actived group exhibited the lowest infiltration of M1 macrophages, which may cause low chemosensitivity. TAMs protect CSCs from chemotherapy and secrete cathepsins that may blunt the chemosensitivity of cancer cells [36, 37]. An agonist CD40 antibody was found to modify macrophages to a tumouricidal phenotype in pancreatic cancer [38]. Moreover, an agonistic CD40 monoclonal antibody (CP-870,893) in combination with gemcitabine showed partial clinical effects in advanced pancreatic cancer patients [39]. Therefore, a CD40 antibody may improve the chemosensitivity of patients in the PD-L1-low-actived group. The results revealed that axitinib showed sensitivity in the PD-L1-low-exhausted group. Combination therapies of immune checkpoint inhibitors with targeted agents have demonstrated both safety and efficacy and have received FDA approval for the first-line treatment of advanced RCC [40]. For their use in the treatment of bladder cancer, more clinical trials are needed. The PD-L1-low-exhausted group showed high infiltration of M2 macrophages and low infiltration of CD8+ T cells. Therefore, the key to transformation to the tumouricidal type is CD8+ T cell proliferation and activation. However, the poor antigen-presenting cell (APC) status of the PD-L1-low-exhausted group leads to inefficient presentation of antigen to CD8+ T cells and includes high infiltration of naïve B cells and low T follicular helper cell and DC activation. T follicular helper cells help B cells differentiate into plasma and memory B cells [41].

In conclusion, to reverse the poor APC status, improving T follicular helper cell and DC activation may be helpful. Treatment with FLT3 L/poly I:C, which induces the activation of CD103+ cDC1s at tumour sites and induce proliferation of naïve CD8 T cells and generated CTLs, has been shown to enhance antitumour responses [42–44].

We performed GSEA to explore the critical pathways in the PD-L1-low-exhausted group. Interestingly, the upregulated mRNAs were enriched in the epithelial mesenchymal transition (EMT) pathway. Recently, Li Wang et al. demonstrated that in metastatic urothelial cancer patients treated with a PD-1 antibody, nivolumab, higher expression of EMT-related genes was associated with lower response rates and shorter PFS and OS. Therefore, cotargeting PD-1 and stromal elements may reverse immune resistance.

The upregulated mRNAs in the other group were enriched in the E2F target pathway. E2F target pathway activation leads to an increase in the levels of proteins required for DNA synthesis, eventually allowing the cell to duplicate itself. For the treatment of other groups, it is well established that the effects of CDK4/6 inhibitors reduce the activity of the E2F target DNA methyltransferase 1 and suppress the proliferation of tumour cells and regulatory T cells [45].

The genome-wide CNV study revealed 381 gene-level areas of gains or deletions. The results revealed that 141 CNV-driven changes in mRNA expression of the 381 CNVs were associated with the PD-L1-low-exhausted group. DEG testing gave a set of 48 gene-level transcripts consistently associated with the PD-L1-low-exhausted group. From these results, the CNV analysis provided translational benefits in DEGs including LCNL1, FBP1 and RASL11B. RASL11B was significant for predicting patient overall survival and disease-free survival. RASL11B is a small GTPase belonging to a Ras subfamily of putative tumour suppressor genes. Upregulated RASL11B inhibits cell proliferation, invasion, and migration, induces G0/G1 cell cycle arrest and promotes cell apoptosis in clear cell renal cell carcinoma (CCRCC) [46]. RASL11B has promise as a therapeutic target.

**Fig. 5:**
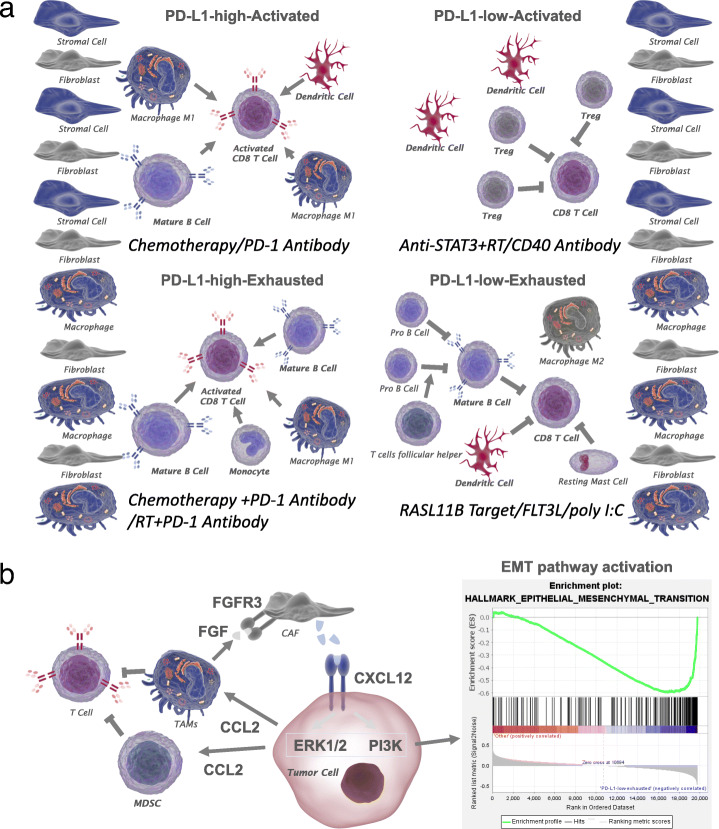
Immune environments and therapeutics used in the immune subgroups and crosstalk in the PD-L1-low-exhausted group. **a** The PD-L1-high-actived group: M1 macrophages and CD8+ T cells highly infiltrated the tumours. It is well established that CD8+ T cells and macrophages are required for synergistic curative activity in response to immune checkpoint antibodies. In addition, high chemosensitivity was also inhibited in this subgroup. The PD-L1-high-exhausted group: The highest infiltration of M1 macrophages and CD8+ T cells and low infiltration of activated dendritic cells. Chemotherapy or RT are needed in combination with immune checkpoint antibodies to improve antitumour responses. The PD-L1-low-actived group: The highest infiltration of Tregs and the lowest infiltration of CD8+ T cells. The high infiltration of activated dendritic cells. In addition, the PD-L1-low-actived group exhibited the lowest infiltration of M1 macrophages, which may cause low chemosensitivity. The CD40 antibody modifies macrophages to a tumouricidal phenotype. STAT3 inhibition in combination with radiation improved tumour growth delay, decreased Tregs and enhanced effector T cells and M1 macrophages, which may improve curative effects. The PD-L1-low-exhausted group: The highest infiltration of M2 macrophages, low infiltration of CD8+ T cells, high infiltration of naïve B cells, low T follicular helper cells and activated dendritic cells. Treatment with FLT3 L/poly I:C, which induces the activation of CD103+ cDC1s at tumour sites and induces the proliferation of naïve CD8 T cells and generated CTLs, has been shown to enhance antitumour responses. In addition, CNV analysis provided translational benefits in the DEG RASL11B, which may be a target for the PD-L1-low-exhausted group. **b** The interactions between tumour cells and stroma mediated through CXCL12 lead to activation of the PI3K and ERK1/2 pathways, which are associated with tumour viability and the active EMT pathway, promoting cell survival and metastasis. CCL2 released by CAFs and tumour cells recruits MDSCs and macrophages. TAMs, which promote the viability of tumour cells, secrete FGF, which binds with FGFR3 and supports CAF survival and activation. Furthermore, TAMs and MDSCs suppress T-cell function in the TME. Abbreviations: CCL2, chemokine (C-C motif) ligand 2; CXCL12, chemokine (C-X-C motif) ligand 12; ERK1/2, extracellular signal-regulated kinase 1/2; FGF, fibroblast growth factor; PI3K, phosphoinosid-3-kinase

In summary, four stable immune subgroups were found in bladder cancer (Fig. 5). These subgroups were found to be associated with prognosis and genetic and immune modulatory alterations that shape immune environments types. Patients in the PD-L1-high-actived and PD-L1-high-exhausted groups were found to have a significantly higher overall survival and better response to chemotherapy and immunotherapy, suggesting that cancer therapeutics can be tailored based on the immune subgroups of the tumour microenvironment. Regarding the lower number of SNV neoantigens in the PD-L1-high-exhausted subgroup, radiotherapy and chemotherapy in combination with anti-PD-1/PD-L1 therapy may be useful in these cases. In the immunologically quiet stage, PD-L1-low-actived patients showed the highest infiltration of Tregs and the lowest infiltration of M1 macrophages and CD8+ T cells and thus may benefit from STAT3 inhibition in combination with radiation or an agonistic CD40 antibody. In addition, pembrolizumab or avelumab with axitinib has demonstrated both safety and efficacy in the first-line treatment of advanced RCC and is recommended for PD-L1-low-actived-type patients, who need more clinical trials. PD-L1-low-exhausted patients showed the worst APC immunosuppression status and had the worst prognosis, and therapeutic approaches targeting RASL11B to improve T cell follicular helper and DC activation (FLT3 L/poly I:C) may be helpful. With an increasing understanding that the immune environment of tumours plays an important role in the response to therapy, defining the immune subtype plays a critical role in predicting disease outcome and strategy effectiveness.

The role of the microenvironment in tumours along with various cell types along with crosstalk was assessed. To elucidate the crosstalk between tumour cells and stroma, we estimated the expression of some factors. Soluble factors secreted by tumour cells and stroma promote angiogenesis and support tumour cell survival. The interactions between tumour cells and stroma mediated through CXCL12 lead to activation of the PI3K and ERK1/2 pathways, which are associated with tumour viability and the active EMT pathway, promoting cell survival and metastasis. CCL2 released by CAFs and tumour cells recruits myeloid-derived suppressor cells (MDSCs) and macrophages. TAMs, which promote tumour cell viability, secrete FGF, which binds with FGFR3 and supports CAF survival and activation. Furthermore, TAMs and MDSCs suppress T-cell function in the TME [12].

Several limitations should be addressed in this study. First, we adopted the CD274 mRNA expression level as a surrogate for PD-L1 expression. Second, the retrospective nature of TCGA causes bias from variations in follow-up information. Furthermore, our findings need to be validated at the protein level.

## Conclusions

In this study, we identified common immune subgroups based on a combination of PD-L1 expression and the type of immune modulation and performed a series of analyses. This suggests the need for prospective studies in immune subgroup patients, especially to better address drug design and understand the outcome of gene-specific therapies.

The PD-L1-low-exhausted group exhibited a worse prognosis than the other groups and showed tumouricidal features (high infiltration of M2 macrophages and low infiltration of CD8+ T cells) and poor APC immune status and activated CAF status. Targeting the immune status of the PD-L1-low-exhausted group would have a tremendous impact on individual treatment.

Copy number variations (CNVs) and differentially expressed genes upregulated in the PD-L1-low-exhausted group, such as RASL11B, played a role in predicting overall survival in TCGA data and was validated in the PD-L1-low-exhausted group in the GEO database. This important result also highlights RASL11B as a therapeutic target, but further investigations are needed.

We believe that reporting data from a large patient cohort might be extremely valuable for many aspects, such as providing patients with a better understanding of tumour immune mechanisms and therapy.

## Data Availability

The datasets generated and/or analysed during the current study are available in the Genomic Data Commons Data Portal (https://portal.gdc.cancer.gov/). The public access to the database is open. The authors did not have special access privileges.
